# Supplementary material for: A self-supervised learning approach for high throughput and high content cell segmentation
Source: Commun Biol. 2025 May 21;8:780. doi: 10.1038/s42003-025-08190-w (PMC12095644; doi:10.1038/s42003-025-08190-w)
Supplement: Supplementary file 1 — Supplementary information [file 42003_2025_8190_MOESM1_ESM.pdf]

Supplementary Note 1

Main Text Figure	Cell Type	Optical Modality	Objective Magnification	Objective Numerical Aperture	Camera
Figure 2a	MDA-MB-231 (human breast adenocarcinoma)	Phase	10X	0.3 (air)	Zeiss Axiocam 702 CMOS
Figure 2b	MDA-MB-231	Bright-field	40X	oil	
Figure 2c.i	MDA-MB-231	DIC	20X	0.8 (air)	Zeiss Axiocam 702 CMOS
Figure 2c.ii, 9a	S. cerevisiae	DIC	63X	1.4 oil	Zeiss Axiocam 702 CMOS
Figure 2d	Hs27 (human foreskin, fibroblast)	IRM	40X	1.4 (oil)	Hamamatsu ORCA R2 CCD
Figure 2e.i,ii	A549 (human lung adenocarcinoma)	Epiflourescence (GFP)	100X	1.46 oil	Hamamatsu ORCA R2 CCD
Figure 2e.iii,iv	MDA-MB-231	Epiflourescence (F-actin and Vinculin)	63X	1.25 (oil)	Photometrics CoolSNAP HQ2 CCD
Figure 3a	Hs27	Epiflourescence (DAPI, F-actin and Vinculin)	20X	0.8 (air)	Zeiss Axiocam 702 CMOS
Figure 3b	Hs27 (human foreskin, fibroblast)	Confocal Flourescence (F-actin and Vinculin)	63X		
Figure 4	Hs27	Epifluorescence (Zeiss3) (Vinculin)	40X	1.4 (oil)	Hamamatsu ORCA R2 CCD
Figure 5, 6a – c (iv-vi)	Hs27	Confocal Flourescence (F-actin)	63X		
Figure 6a – c (i-iii)	Hs27	Epiflourescence (F-actin)	10X	0.3 (air)	Zeiss Axiocam 702 CMOS
Figure 6a – c (iv-vi)	Hs27				
Figure 8a-b	Hs27	Epiflourescence (Vinculin)	10X	0.3 (air)	Zeiss Axiocam 702 CMOS

Supplementary Note 2: Self-supervised learning segmentation approach before and after applying declumping step

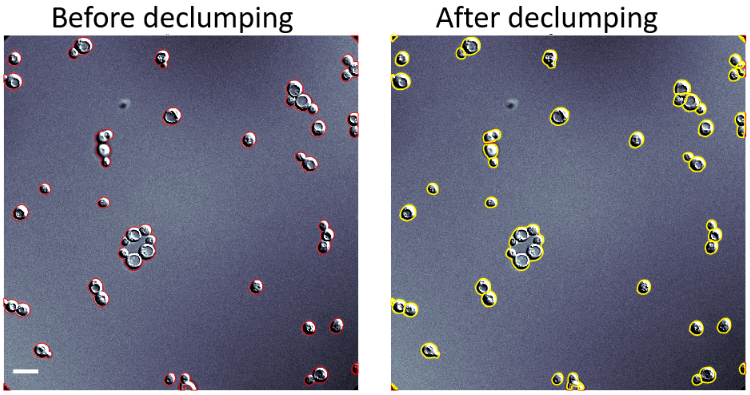

Figure: Self-supervised learning segmentation approach on S. cerevisiae image taken by DIC (63X objective) with and without declumping step. Scale bar: 5µm

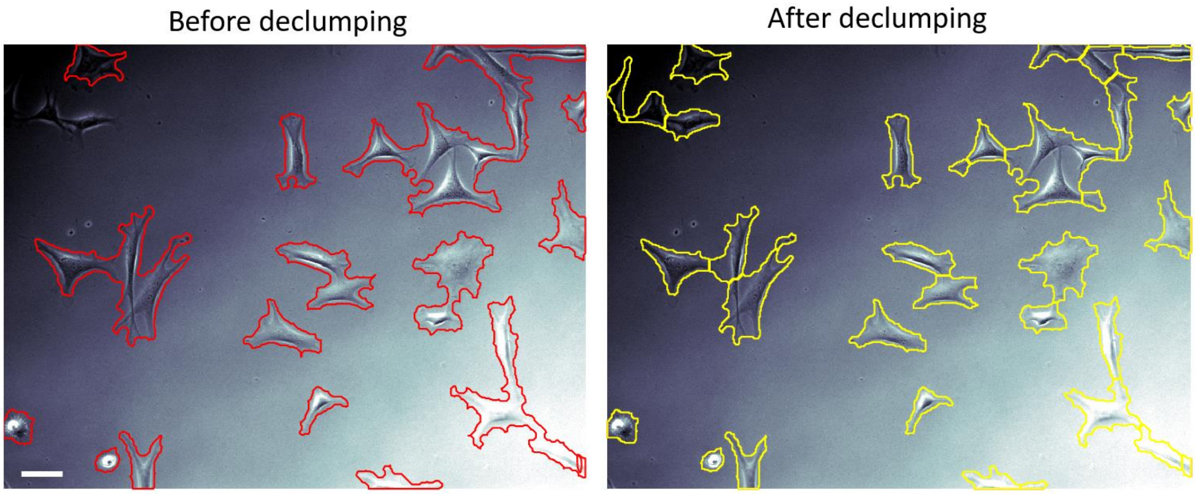

Figure: Self-supervised learning segmentation approach on Hs27 image taken by phase microscopy (10X objective) with and without declumping step. Scale bar: 50µm

Supplemental Note 3: **Summary of segmentation performance by SSL and different Cellpose models on several imaging datasets.**

	Model	% of exported segmentation images	F1 score	Time (minutes)
**Epifluorescence Hs27 60X** Number of images = 67	SSL	100%	**0.758**	19.03
	cp cyto (d=50)	82.09%	0.078	12.07
	cp_cyto (d=100)	49.25%	0.119	8.33
	cp_cyto (d=200)	38.81%	0.192	9.47
	cp_cyto (d=300)	29.85%	0.253	8.11
	cp_cyto (d=400)	35.82%	0.339	13.73
	cp_cyto2 (d=50)	100%	0.059	12.54
	cp_cyto2 (d=100)	85.07%	0.195	10.58
	cp_cyto2 (d=200)	82.09%	0.455	15.64
	cp_cyto2 (d=300)	68.66%	0.674	26.83
	cp_cyto2 (d=400)	62.69%	0.608	33.80
	cp_cyto3 (d=50)	86.57%	0.072	14.78
	cp_cyto3 (d=100)	70.15%	0.191	17.21
	cp_cyto3 (d=200)	74.63%	0.337	36.2
	cp_cyto3 (d=300)	67.16%	0.400	43.95
	cp_cyto3 (d=400)	53.73%	0.454	38.63
**Epifluorescent Hs27 10X** Number of images = 20	SSL	100%	0.8877	10.49
	cp_cyto2 (d = 50)	100%	0.795	11.05
	cp_cyto2 (d = 100)	100%	0.882	16.67
	cp_cyto2 (d = 150)	100%	0.792	16.56
**Phase Contrast Hs27 10X** Number of images = 565	SSL	100%	0.771	129.33
	cp_cyto2 (d = 30)	100%	0.622	142.00
	cp_cyto2 (d = 50)	100%	0.777	148.05
	cp_cyto2 (d = 100)	100%	0.614	118.7
**Epifluorescence Nucleus** Number of images = 189	SSL	100%	0.873	23.47
	cp_nuclei (d = 30)	100%	0.953	58.01
	SSL + cp_nuclei (d=30)	100%	0.944	76.03
	SSL + declumping	100%	0.873	24.22
**Epifluorescence Hs27 40X** Number of images = 17	SSL	100%	0.831	3.02
	cp_cyto2 (d=150)	82.35%	0.818	5.154
**DIC S. cervia 63X** Number of images = 60	SSL	100%	N/A	36.35
	yeast_PhC_cp3 (d= 50)	100%	N/A	65.67
	SSL + declumping	100%	N/A	39.14
